# Effects of Positive End Expiratory Pressure On Regional Ventilation-Perfusion Matching And Respiratory Mechanics: A Clinical Study

**DOI:** 10.1186/2197-425X-3-S1-A8

**Published:** 2015-10-01

**Authors:** T Mauri, N Eronia, Turrini, BC Cambiaghi, A Benini, G Bellani, A Pesenti

**Affiliations:** IRCCS “Ca' Granda Foundation, Maggiore Policlinico Hospital, Milan, Italy; University of Milan-Bicocca, San Gerardo Hospital, Monza, Italy; University of Ferrara, Sant'Anna Hospital, Ferrara, Italy

## Introduction

In intubated acute respiratory failure patients, inhomogeneity of ventilation-perfusion matching (i.e., presence of over-ventilated under-perfused lung regions) might determine extreme pH shifts and increase the risk of developing ventilator-induced injury. Positive end-expiratory pressure (PEEP) redistributes tidal ventilation towards more perfused dependent lung regions, potentially improving homogeneity of ventilation-perfusion matching.

## Objectives

In this study, we used Electrical Impedance Tomography (EIT) to assess global and regional lung ventilation and perfusion at different PEEP levels and to verify the effects of PEEP on homogeneity of ventilation-perfusion matching.

## Methods

We enrolled 20 intubated critically ill patients undergoing controlled mechanical ventilation, sedated, paralyzed, with PaO_2_/FiO_2_≤300 mmHg and PEEP≥5 cmH_2_O. We randomly applied two PEEP levels (clinical and clinical + 5 cmH_2_O) for 20 minutes each and collected ventilation and EIT data at the end of each step. From EIT, we measured: 1. regional ventilation heterogeneity (VtHet, defined as the ratio between Vt reaching non-dependent/dependent lung); 2. regional homogeneity of ventilation-perfusion matching (H_V/P_); 3. regional compliance; 4. cumulated regional lung hyperdistension.

## Results

**Figure 1:**
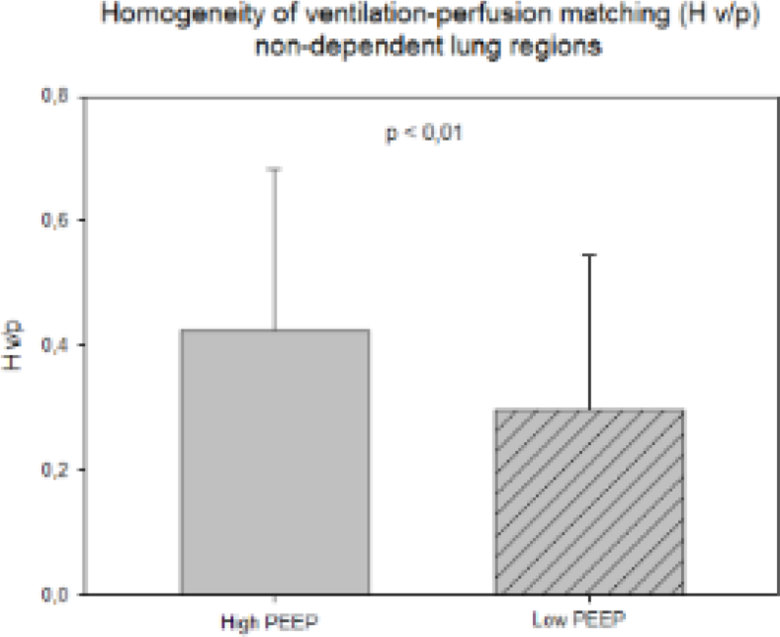
**H v/p non-dependent regions.**

**Figure 2:**
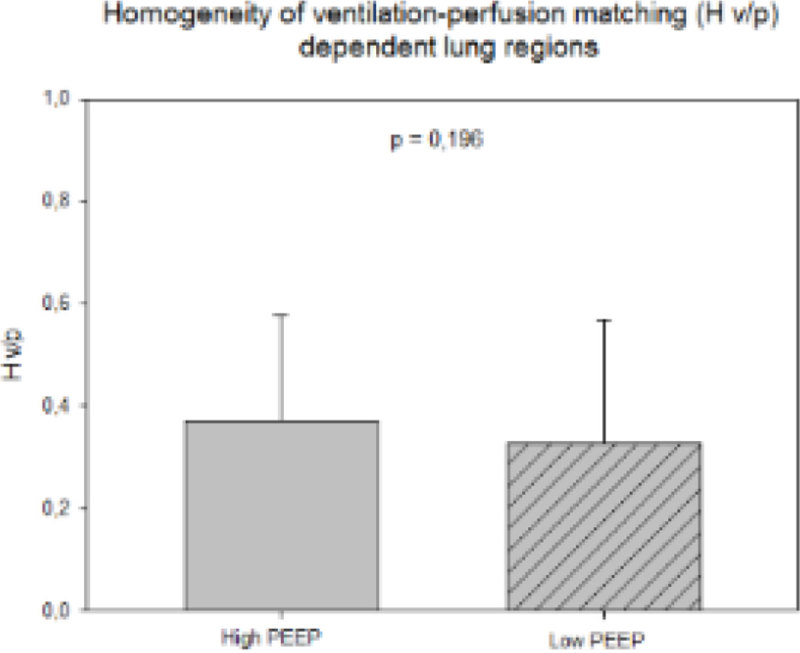
**H v/p dependent regions.**

Patients were 62 ± 12 years old, PaO_2_/FiO_2_ was 197 ± 52 mmHg, lower PEEP was 7 (7-9) cmH_2_O while higher PEEP was 12 (12-14) cmH_2_O (p < 0.001). At higher PEEP, VtHet was reduced (1.8 (1.5-2.4) vs. 2.2 (1.8-2.6), p < 0.001). Regional H_V/P_ improved at higher PEEP in non-dependent areas (0.42 ± 0.24 vs. 0.29 ± 0.25, p < 0.01) as well as in the dependent ones, albeit non-significantly (0.37 ± 0.20 vs. 0.33 ± 0.24, p = 0.196) (Figures 1 and 2).

Finally, by applying higher PEEP, regional compliance of non-dependent lung decreased (31 ± 12 vs. 37 ± 13 mL/cmH_2_O, p < 0.001) and cumulative hyperdistension of the same areas significantly increased (+18 ± 7%, p < 0.001).

## Conclusions

Improved homogeneity of ventilation-perfusion matching might represent one of the protective mechanisms associated with the use of higher PEEP. On the other hand, such benefits must be balanced with increased risk of hyperdistension of non-dependent lung.

## Grant Aknowledgement

Institutional.

